# Exploring the Association Between the “Big Five” Personality Traits and Fatal Opioid Overdose: County-Level Empirical Analysis

**DOI:** 10.2196/24939

**Published:** 2021-03-08

**Authors:** Zhasmina Tacheva, Anton Ivanov

**Affiliations:** 1 School of Information Studies Syracuse University Syracuse, NY United States; 2 Department of Business Administration Gies College of Business University of Illinois at Urbana-Champaign Urbana-Champaign, IL United States

**Keywords:** opioid addiction, personality traits, community health, text mining, opioid, addiction, psychological

## Abstract

**Background:**

Opioid-related deaths constitute a problem of pandemic proportions in the United States, with no clear solution in sight. Although addressing addiction—the heart of this problem—ought to remain a priority for health practitioners, examining the community-level psychological factors with a known impact on health behaviors may provide valuable insights for attenuating this health crisis by curbing risky behaviors before they evolve into addiction.

**Objective:**

The goal of this study is twofold: to demonstrate the relationship between community-level psychological traits and fatal opioid overdose both theoretically and empirically, and to provide a blueprint for using social media data to glean these psychological factors in a real-time, reliable, and scalable manner.

**Methods:**

We collected annual panel data from Twitter for 2891 counties in the United States between 2014-2016 and used a novel data mining technique to obtain average county-level “Big Five” psychological trait scores. We then performed interval regression, using a control function to alleviate omitted variable bias, to empirically test the relationship between county-level psychological traits and the prevalence of fatal opioid overdoses in each county.

**Results:**

After controlling for a wide range of community-level biopsychosocial factors related to health outcomes, we found that three of the operationalizations of the five psychological traits examined at the community level in the study were significantly associated with fatal opioid overdoses: extraversion (β=.308, *P*<.001), neuroticism (β=.248, *P*<.001), and conscientiousness (β=.229, *P*<.001).

**Conclusions:**

Analyzing the psychological characteristics of a community can be a valuable tool in the local, state, and national fight against the opioid pandemic. Health providers and community health organizations can benefit from this research by evaluating the psychological profile of the communities they serve and assessing the projected risk of fatal opioid overdose based on the relationships our study predict when making decisions for the allocation of overdose-reversal medication and other vital resources.

## Introduction

### Background

According to the Centers for Disease Control and Prevention (CDC), “Opioid abuse and overdose deaths are at epidemic levels in the United States” [1] and are now outpacing car accident fatalities [2]. To address this crisis, government agencies, health care providers, and university researchers alike have considered both big data and technological innovation as sources of solutions. Ingestible sensors monitoring opioid intake, tracking opioid dispensing rates, and pairing electronic health records with e-prescribing data [3] are some of the promising ways that information systems can help advance medical understanding and action in the context of a fatal opioid overdose. Although existing opioid overdose programs such as those providing Naloxone address the problem in a reactive fashion (ie, when the patient has already taken a nearly fatal dose of medication), this study proposes an approach that could allow health care providers and officials to act proactively in a preventive manner (eg, by prescribing higher-schedule drugs to people in higher risk categories). In particular, we seek to model fatal overdose by taking into account psychological and behavioral traits that often assume the role of invisible underlying factors. As our theoretical foundation, we use the five-factor model (FFM) of the “Big Five” personality traits. Its dimensions are often referred to as OCEAN (openness, conscientiousness, extraversion, agreeableness, and neuroticism). OCEAN and their relationship to substance use has been studied extensively in both the medical and psychological literature [4].

The effect of the FFM dimensions on substance use has been demonstrated across different contexts, including but not limited to age and gender groups [5,6], nationalities [7,8], length and intensity of use [9,10], and types of substance [11,12]. Contextual differences notwithstanding, research shows that personality traits represent a significant factor in understanding various types of substance use, including opioids [10,13]. The effects of the personality traits have been fairly consistent and stable in predicting different aspects of substance use [4] (except for extraversion, which has shown less clear and often inconsistent results). Keeping the relationship between opioid use and personality in mind, we focus on a nascent stream of the FFM inference literature, which emphasizes the feasibility of inferring the Big Five personality traits from self-expressive written artifacts such as social media posts [14,15] due to the moderate to high correlations between the linguistic features of such social media messaging and personality trait measurements established through conventional psychological test surveys [16]. To this end, our study uses extensive unstructured data available from Twitter (we collected and analyzed nearly 19 million geo-tagged tweets) in combination with a literature-driven linguistic analysis approach [17] to derive unique personality profiles on US counties (known as geo-personality).

The potential of social media content for epidemiological surveillance has been demonstrated in the cases of influenza [18] and HIV [19], as well as in the context of adverse behaviors such as suicide [20] and drug abuse [21]. Unlike traditional methods of epidemiology and surveillance (which require significant time and resources to collect and analyze medical diagnostic information, thereby increasing the gap between emergency and response), social media surveillance offers quicker detection and response [22]. Among social media platforms, Twitter has emerged as the leading source of digital surveillance data. In particular, the level of granularity of its data coupled with the ease of data retrieval through the official application programming interface make it feasible to integrate the spatial, temporal, and text models into a unified framework for detection [23].

To ensure the reliability and consistency of our model, which seeks to explore the relationship between personality traits inferred from social media text data and fatal opiate overdose, we used an extensive set of control variables identified by prior literature and relied on a rigorous econometric specification. To alleviate the endogeneity concerns caused by the omitted variable bias, we used a control function approach.

Our analysis yields several important results that illustrate the potential of social media surveillance for improving drug safety and offer theoretical and practical implications for improved patient care, public health, and well-being. Specifically, this study demonstrates the feasibility of assessing the Big Five personality traits from user-generated online content at scale in real time and extends the health informatics literature on the association between personality and opioid fatality, which has thus far only explored this relationship at the state level [24], to a more granular, county-level context. Our results are largely consistent with medical and psychological theory: we find that the traits of extraversion and neuroticism have a significant positive impact on the number of opioid deaths. A divergent finding is the positive effect of conscientiousness on opiate mortality, which persists even with an alternative data mining personality trait inference technique, thereby pointing to the need for a critical examination of the extant computational methods for personality assessment. This surprising outcome notwithstanding, the study shows that personality is a factor that cannot be ignored in the analysis of opioid use behaviors and provides an effective way to infer and integrate it into a comprehensive yet easy to implement model.

### Literature Review

Opioids are a class of psychoactive medicinal substances that include semisynthetic prescription pain relievers, synthetic opiates such as methadone and fentanyl, and the illicit drug heroin. Opioids interact with opioid receptors on nerve cells in the brain and nervous system to produce pleasurable effects and relieve pain [25]. Unfortunately, these beneficial effects are often outweighed by the risk of opioid drug dependency—a treacherous path toward addiction and possibly death.

The first stream of literature related to our study focuses on the factors contributing to fatal and nonfatal opioid overdose. Looking through the prism of the *biopsychosocial* model of health and disease proposed by Engel [26], these factors can be broadly categorized as *biological* (age, gender, and comorbidity [27]; history of substance use disorders [28]; or medication intake [29]), *psychological* (sexual identity [30], sexual behavior [31], and history of psychiatric problems [32]), and *socioeconomic* (socioeconomic status [33], educational attainment [28], and history of criminal charges and detention [34]). Despite these three factors’ long history in medical research on opioid overdose, research in to personality’s influence on fatal drug overdose is lacking.

A second stream of literature examines the role of personality in health care decision making. Specifically, psychology has assembled a compelling body of evidence in support of the link between personality and health behaviors and outcomes [35]. In longitudinal studies, the best-known taxonomy of personality, the “Big Five” Factor model, has been found to be predictive of health care decision-making styles [36], physician visits and hospitalization probability [37], longevity [38], and obesity [39], among other things.

In the context of substance use specifically, a rich body of literature in both the medical and psychological domains has amassed ample evidence of the relationship between the five FFM traits and various aspects of substance use and dependence. To facilitate comprehension of the role (positive, negative, or insignificant) of each personality trait in substance use established in the extant literature, we provide Table 1. We further use information inferred from this table in the hypotheses development section that follows.

**Table 1:** The effects of “Big Five” personality traits in the context of substance use.

Study	Substance	Openness	Conscientiousness	Extraversion	Agreeableness	Neuroticism
[40]	Lifetime diagnosis of substance abuse or dependence (including nicotine)	+^a^	–^b^	–	–	+
[41]	SCID^c^ substance dependence severity; polydrug use; alcohol	+	–	–	–	+
[42]	Male substance-abusing veterans	N/A^d^	–	N/A	–	+
[43]	Cocaine, alcohol, and heroin	N/A	–	N/A	–	+
[12]	Alcohol abuse	N/A	–	+	N/A	N/A
[12]	Marijuana abuse	+	N/A	–	N/A	N/A
[44]	Alcohol and drug dependence	N/A	–	+	–	N/A
[5]	Youth with conduct and substance use disorders	N/A	–	N/A	–	+
[13]	Opioid dependence	N/A	–	–	N/A	+
[11]	Tobacco, cocaine, and heroin use	N/A	–	N/A	N/A	+
[11]	Marijuana use	+	–	N/A	–	+
[45]	Substance abuse	–	–	+	N/A	+
[46]	Alcohol use	N/A	–	+	–	N/A
[9]	Longitudinal substance use, including tobacco, alcohol, and illicit drugs	+	–	+	–	+
[47]	Nonmedical prescription drug use in young adults	+	–	N/A	N/A	+
[10]	Longitudinal pain and prescription opioid medication use	–	N/A	–	N/A	+
[48]	First-time and subsequent illicit drug use	+	–	+	–	+
[6]	Alcohol use disorder	N/A	N/A	–	–	N/A
[6]	Drug use disorder	N/A	N/A	N/A	–	N/A

^a^Indicates a positive effect.

^b^Indicates a negative effect.

^c^SCID: Structured Clinical Interview for Diagnostic and Statistical Manual of Mental Disorders IV.

^d^N/A: not applicable.

Despite these compelling findings, there is, to the best of our knowledge, a dearth of research exploring personality’s role in fatal opioid overdose behavior specifically.

A methodological commonality between the aforementioned streams of literature is their preferred research design: experimental or quasi-experimental cross-sectional or longitudinal cohort studies. Although this design is the “gold standard” for establishing internal validity, health care researchers have long emphasized the need for increased generalizability (ie, external validity) of research findings [49]. Relatedly, new research opportunities provided by the Big Data analytics suggest an avenue for enhancing generalizability through the analysis of unstructured social data at the population level, as opposed to a limited group of individuals [50]. This analytical approach is justified due to the well-documented intrapersonal stability of Big Five traits [51] and the established feasibility of capturing population psychological characteristics through social media [52]. Specifically, the Big Five personality trait scores predicted using psycholinguistic computational modeling have been shown to moderately (.48) to strongly (.65) correlate with the ground-truth personality measurements obtained through personality questionnaires [16]. The mean absolute error of the scores predicted by this psycholinguistic approach was approximately 11% for each personality trait, suggesting that personality inference based on user-generated text can detect a trait to within slightly more than a tenth of its actual value [16]. Therefore, to address the gap created by the lack of studies investigating the link between personality traits and opioid fatalities at the community (county) level, our study uses a novel and reliable methodology that relies on an expansive survey of social data from the majority of counties in the United States. Our research question, then, is “How can we use the Big Five personality traits in mitigating the opioid overdose crisis?”

Our investigation of this research question contributes to health informatics by demonstrating the feasibility of intelligently mining unstructured (Twitter) data for epidemiologic discoveries. In particular, we make a theoretical contribution by elucidating the relationship between personality and health-related outcomes. Specifically, we provide a more nuanced understanding of personality’s influence on fatal opioid overdose through the five distinct dimensions of the five-factor personality trait model. To do so, we build on a burgeoning stream of health care informatics, which establishes social media posts on the topic of opioid substances as a timely indicator of opioid overdose mortality [24,53], by using a combination of advanced computational techniques (cloud computing and text mining) and robust econometric analysis to expand the scope of user-generated content relevant to infoveillance beyond posts directly mentioning opioids. Our study also has several practical implications for health care providers and administrators, as its findings can be applied in opioid overdose prevention and surveillance based on the local counties’ prevalent personality traits.

### Hypotheses Development

The principal theoretical foundation for this paper derives from the extensive body of research on personality traits. *Personality traits* are enduring styles of thinking, feeling, and acting that characterize an individual [54]. The relative stability of these traits points to consistent and recurrent patterns of acting and reacting that both characterize individuals and differentiate them from others. Similarly, they lead to empirical generalizations about how people with similar traits are likely to act and react [55]. Personality traits have consistently been shown to influence a wide variety of interests and behaviors, such as vocational, social, and artistic interests [54]; brand trust and affect [56]; and internet use [57]. Furthermore, in the health care context, the robust predictive capacity of the personality traits has been established in such complex behaviors as alcohol consumption, exercise routine and obesity index [58], smoking and BMI [59], overall substance use [60], and general health and functional status [59]. The strong link between personality traits and human behaviors, which makes possible the extrapolation of potential future behavioral outcomes based on a given set of personality traits, warrants an in-depth investigation of personality’s impact on opioid overdose patterns. Specifically, we used the “Big Five” FFM of personality, considered the most robust categorization of personality traits to date [61]. Notably, the Big Five have demonstrated to be universally representative and to exhibit the same structure across different regions and cultures [62].

The use of the FFM in the study of opioid overdose is particularly salient because of the long-standing stream of studies exploring its relationship with various substance use behaviors, summarized in Table 1. As the table shows, all five traits have a statistically significant effect on substance use, documented across studies spanning different research settings, such as age and gender groups [5,6], nationalities [7,8], length and intensity of use [9,10], and types of substance [11,12]. As evident from the findings of prior studies, different personality traits in the FFM framework play a different role in substance-related behaviors. We further formulate a set of testable hypotheses informed by the extant literature.

*Openness* is characterized by a high degree of intellectual capacity, wide interests, and unconventional thought [63]. Meta-analyses of the relationship between the Big Five and substance use disorders have largely failed to find a significant impact of openness on substance abuse [4] and mental illness [64]. However, multiple individual studies have found a statistically significant relationship between openness and various types of substance use. Only two studies to date have documented a negative effect of openness on substance use [10,45], while the majority have established a positive effect for the following behavioral constructs: substance abuse and dependence [40,41], marijuana use [11,12], first-time and subsequent illicit drug use [48], and longitudinal drug use [9]. Since the overwhelming majority of FFM studies on substance use point to a positive role for openness, we hypothesize the following:

- Hypothesis 1: Openness will have a positive impact on fatal opioid overdose.

*Conscientiousness* combines the traits of being diligent, thorough, and being governed by one’s conscience [65]. It has a negative relationship with mental illness [64] and various substance use disorders [66]. Specifically, conscientiousness has a known negative effect on alcohol abuse and dependence [12,44,46], longitudinal substance use [9,40], and drug use in particular [11,48]. In addition to the consistent and robust findings in this domain, high scorers on this dimension are expected to shun intentional overdose due to imminent feelings of guilt and this trait’s strong underlying facets of responsibility, traditionalism, and self-control [67]. This leads us to hypothesize the following:

- Hypothesis 2: Conscientiousness will have a negative impact on fatal opioid overdose.

*Extraversion* is characterized by positive affectivity, adventurousness, energy, warmth, and gregariousness [65]. Although this trait has been found to be negatively associated with psychopathology (eg, depression and anxiety) [68] and higher levels of extraversion have been associated with better self-rated health [69] and greater physical activity [59], its role in substance use behaviors remains unclear in the literature [9]. This lack of clarity is evident from the inconsistent empirical findings for this personality indicator—a phenomenon not observed for the other four traits. In particular, some studies found a positive relationship between extraversion and substance use [44,48], while others document a negative one [13,41] or do not detect an effect [5,11]. Some studies found opposite effects for different substances, namely, a positive effect for alcohol abuse but a negative one for marijuana abuse [12], but it is also possible to detect opposing effects even for the same substance, as in the case of alcohol use disorder, dependence, and abuse, which is positively associated with extraversion in some studies [12,44] but negatively in others [6]. In light of these conflicting findings and the well-documented lack of consistency in this indicator’s effect on substance use, we contend that when it comes to fatal intake of opioids, the role of extraversion is best captured in a set of competing hypotheses. We therefore hypothesize the following:

- Hypothesis 3a: Extraversion will have a positive impact on fatal opioid overdose.
- Hypothesis 3b: Extraversion will have a negative impact on fatal opioid overdose.

*Agreeableness* comprises traits such as trust, modesty, compliance, caring, and emotional support [65]. It is negatively associated with substance use [70], substance dependence severity and polydrug use [41], lifetime substance abuse or dependence [40], alcohol and drug dependence [44], marijuana use [11], cocaine and heroin use [43], first-time and subsequent illicit drug use [48], and substance use and addictive disorders [6,71]. In keeping with the extant literature, we hypothesize the following:

- Hypothesis 4: Agreeableness will have a negative impact on fatal opioid overdose.

*Neuroticism* (also referred to as emotional range) is reflected both in a person’s tendency to experience distress and in the cognitive and behavioral styles that stem from it. Individuals scoring high on this dimension tend to experience chronic negative effects and are prone to various psychiatric disorders [65]. Neuroticism has a strong positive relationship with mental illness, anxiety disorders, internet addiction, smoking, distress, and internalizing problems [72]. Moreover, several studies have found a positive relationship between neuroticism and substance use disorders [4,70,72], opioid abuse [73], and nonmedical prescription drug use [47,74]. Perhaps most telling of this trait’s potential role in opioid overdose is its documented positive effect on longitudinal pain and prescription opioid medication use [10]. Death due to opioid overdose can be viewed as another facet of the inherent risk of self-harm associated with the depressive states characteristic of neuroticism. We therefore hypothesize the following:

- Hypothesis 5: Neuroticism will have a positive impact on fatal opioid overdose.

## Methods

### Mortality Data

The first step in our data collection is related to the dependent variable: opioid overdose deaths. These yearly (2014-2016) panel data were obtained through the WONDER (Wide-Ranging Online Data for Epidemiologic Research) online database [75] from the CDC. This is the primary (and only) publicly available source that provides mortality data based on underlying cause of death, especially at the *county* level. Data are based on death certificates for US residents. Each death certificate contains a single underlying cause of death (and as many as 20 additional contributing causes) and demographic data.

Importantly, due to confidentiality constraints enforced by the CDC, all subnational data points representing zero to nine deaths or births are suppressed [76]. Given this constraint, our sample includes complete mortality data on 701 out of 3007 counties in the United States. To get a sense of opioid-related deaths across counties by year, consider the descriptive statistics in Table 2. To address the limitations associated with data suppression, we used appropriate econometric modeling techniques (discussed later in this paper).

**Table 2:** Descriptive statistics of the number of opioid-related deaths.

Year	Nonsuppressed counties (>9 deaths), n	Observed number of deaths per county, mean	Observed total deaths across all counties, n	Actual^a^ total deaths across all counties, n
2014	585	41	23,923	28,647
2015	617	46	28,185	33,091
2016	701	54	37,526	42,249

^a^Although data on counties with fewer than 10 individuals affected were not available, we were able to obtain data on the total number of deaths across all counties. We subtracted the number of known opioid-related deaths from the total deaths and then divided the result by the “suppressed” counties’ populations. The resulting (approximated) mean number of deaths in the suppressed counties equaled 0.8 (SD 1.7).

### Twitter Data

In the second step, we obtained unstructured text data for language analysis from Twitter and integrated it with the mortality data. For the purpose of our analysis, we used the publicly available snapshots of Twitter traffic known as “spritzer.” This type of Twitter grab provides a vast volume of data for incisive analysis. For example, consider the structure of a single monthly data archive (file) that was preprocessed for text mining purposes: January → 31 days → 24 hours → 60 minutes → 1 minute → *JavaScript Object Notation* (*JSON*) *file.* Each single JSON file contains 1% of Twitter traffic grabbed in a given minute. Each monthly archive (about 450 GB) contains 43,800 (ie, the number of minutes in a month) JSON files with Twitter data (tweets). Extensive data collection and preprocessing (of nearly 17 TB of text data) was accomplished by means of powerful cloud computing resources provided by Amazon Web Services.

Notably, for the purpose of our analysis, we extracted only those tweets that were in English and included a geo-tag (metadata with information on the latitude and longitude associated with the location where the tweet originated). Having preprocessed 36 months of data, we were able to extract nearly 19 million tweets satisfying the aforementioned requirements. Given the structure of the spritzer data set, we found no two tweets that originated from the same account. In other words, the almost 19 million tweets used in our analysis represent unique accounts. Next, we excluded duplicate tweets that were posted by the same author and those that contained less than three words (such tweets accounted for approximately 2% of the whole data set). Further, for the purpose of our county-level analysis, we linked tweets to their origins in the respective counties in the United States. To accomplish this, we linked the geographic coordinates contained in the geo-tags to the respective county Federal Information Processing System (FIPS) codes using the -*geoinpoly*- module [77] for Stata statistical package.

Finally, to increase the validity and reliability of our personality mining approach (which is dependent on the volume of text used for mining), we created personality profiles of the individual counties (vs individual tweets at the user level) by aggregating (ie, concatenating) the resulting text data extracted from tweets at the FIPS code level by year. The resulting mean number of words was about 7000 (SD 11,000).

Given that our final sample includes approximately 18.7 million unique users, our sample represents approximately 26% of the total number of Twitter users in the United States (about 69 million). It also represents approximately 6% of the US population (about 316 million in 2013-2014).

It shall be noted that the actual origin of the tweet might not necessarily have a relationship with that county’s incidence. For example, a person might reside in one county (eg, a rural one) but receive a diagnosis or treatment in another county (eg, an urban one); in this case, it would be unclear in which county the tweet actually originated. Therefore, to ensure the robustness of our assumption that tweets in our sample originated from the corresponding counties, we conducted the following analysis. First, we identified those users who self-reported their “location” in their Twitter profiles; they represented approximately 7% of the sample. Second, we compared the “location” value with the tweet origin (as indicated by the geo-tag). The result showed that, of the 7% of users who specified their location, almost 98% tweeted from the same geographic location. These findings confirmed the plausibility of our assumption that the vast majority of the tweets in our sample originated from the counties where the Twitter users in our sample resided.

To ensure that we had approximately equal amounts of data from different types of counties (ie, rural vs urban), we converted the FIPS codes identifying the counties in our sample to the National Center for Health Statistics Urban–Rural Classification Scheme and then examined the distribution of tweets (in terms of word count, because number of individual tweets is a weaker approximation due to the varying number of characters, which range from 1 to 140) across six categories of urban–rural classification (1=large central metro; 2=large fringe metro; 3=medium metro; 4=small metro; 5=micropolitan; 6=noncore). Our results suggest a relatively equal split of the data across all six categories except for the noncore counties, where the total number of tweets is lower due to sparse populations.

### Population Characteristics Data

In the final step of data collection, we merged the opioid-related mortality data and Twitter data with an extensive set of county-level population characteristics provided by County Health Rankings and Roadmaps (CHRR). The CHRR program is a collaboration between the Robert Wood Johnson Foundation and the University of Wisconsin Population Health Institute, which provides granular yearly (2014-2016) panel data on health outcomes and behaviors, clinical care, social and economic environments, and physical environments for the more than 3000 US counties [78]. Using a combination of Twitter data along with population characteristics (including those related to health) has been used in a multitude of recent studies [64,79,80].

### Dependent Variable

The dependent variable in our study is the *number of deaths associated with opioid drug overdose*. When selecting the underlying causes of death for this variable (based on the recommendations provided by a CDC WONDER official representative in a personal communication), we used the number of deaths for the following International Statistical Classification of Diseases and Related Health Problems, 10th revision (ICD-10) codes: T40.0 (opium), T40.1 (heroin), T40.2 (other opioids), and T40.3 (methadone). These data included the following underlying cause of death classifications: drug/alcohol-induced causes: drug poisonings (overdose) unintentional (X40–X44); drug poisonings (overdose) suicide (X60–X64); drug poisonings (overdose) homicide (X85); and drug poisonings (overdose) undetermined (Y10–Y14). As noted previously, the data for counties with fewer than 10 deaths were *suppressed*; that is, the data were not available to the public under any circumstances due to the CDC’s privacy policy.

Such a limitation imposes a substantial constraint on the number of observed counties for which data are available (approximately 23% of all US counties), negatively influencing the generalizability of our analysis and findings. One way to address this issue is to impute the missing values using a state-level opioid-related death rate [81] and treat them as left-censored data. However, an even more advantageous approach that relaxes the underlying assumptions on censoring is to treat our outcome as an interval. That is, because the number of deaths cannot be negative, we can treat the missing observations in the outcome as an interval censored between zero and nine. Therefore, to model our dependent variable, we used an interval regression approach. To account for possible limitations associated with our imputation approach and ensure consistency of our estimates, we also ran a fixed-effects model on the reduced sample (see Table B1, Multimedia Appendix 1).

### Independent Variables: Personality Traits Mining

Although the analysis of personality traits constitutes an important facet of our understanding of opiate addiction and recovery, measuring latent personality characteristics is a challenging process [17]. Particularly, traditional personality trait inference involves conducting in-depth personality tests and surveys—a resource-intensive task that is not easily scalable [15]. Such analysis becomes even more complicated when the goal is to assess personality traits of population *groups* (eg, communities, counties, or states) versus individuals.

Computational advances over the past decade have, however, presented an alternative approach that relies on widely available data sources including user-generated content. Specifically, it has been shown that the language one uses, which can be retrieved from their blog posts or other social media messages, is linked to their unique psychological profile [14,82]. This makes possible the use of unstructured text processing methods for assessing personality traits in a reliable and scalable way [83]. Indeed, recent studies have not only demonstrated the feasibility of a lexicon-based approach for personality trait inference but have also shown that this approach is comparable in its effectiveness to the traditional survey-based personality assessment approach and able to predict actual personality traits to within nearly a tenth of their true values [15,16]. Following this promising approach, we adopted a robust lexicon-based implementation well established in the information systems literature [17,84]. We operationalized our main predictor variables—OCEAN—by analyzing a vast unstructured body of tweets obtained from Twitter. Tweets (short messages) represent a form of user-generated content in which individuals’ written speech samples might contain a variety of psychological, emotional, cognitive, and structural components that can provide clues to these characteristics. To extract information on the Big Five personality traits associated with individual US counties, we used tweets aggregated (concatenated) at the county level and merged into a single vector per county to infer the latent personality traits by means of linguistic analysis [24]. Specifically, after a preprocessing step including stop word removal, stemming, and lemmatization, the content of each vector was matched with the Linguistic Inquiry and Word Count (LIWC) psycholinguistic dictionary, which had undergone several iterations and presently contains more than 90 distinct variables grouped into categories, including 41 categories capturing psychological constructs such as affect, cognition, and drives [85].

Once the LIWC linguistic dimensions for each vector were available, we followed the procedure in Adamopoulos et al [17] and matched them with their corresponding weighted coefficients developed by Yarkoni [14] by estimating the relationships between OCEAN dimensions obtained from traditional psychological test assessments and LIWC items from user-generated content by the same individuals. The product of each LIWC item score and its corresponding weighted coefficient was used to calculate the dot product for each OCEAN trait for each county, which was then rendered as a percentile score to obtain a comparable indicator for each psychological trait across counties [17,86].

This method for personality trait inference has several important advantages over both traditional psychological test assessments and other self-expression approaches. Compared to traditional survey-based inference, it does not burden respondents with lengthy tasks that are sometimes prohibitive, hamper scalability, and are prone to social-desirability bias whereby the respondent might provide answers about an ideal self rather than their actual character [17]. On the other hand and unlike other self-expression methods, which often include the analysis of writing samples collected in laboratory settings, the analysis of social media data does not impose any restrictions on the length or topic of the writing sample, which makes it more naturalistic and able to more fully reveal underlying personality traits [14].

### Control Variables

To ensure correct identification of the focal effects, we included an extensive set of county-specific control variables associated with the health and well-being of the counties’ populations (Table 2). Furthermore, based on the prior literature, we considered two essential correlates of the Big Five: *alpha* and *beta* “superordinate” (high-order) factors [61,87]. These factors are based on the *facets* that underlie the corresponding personality traits. Numerous replications have confirmed the correspondence of alpha, or stability, to neuroticism, conscientiousness, and agreeableness, and that of beta, or plasticity, to extraversion and openness [88,89]. To extract data on a plethora of personality facets underlying the corresponding alpha and beta dimensions, we used the IBM Watson Personality Insights service, a tool used in prior studies [90]. (We provide relevant descriptive statistics and operationalizations in Table B1, Multimedia Appendix 1.)

To account for the reflective nature [89] of alpha and beta, we employed principal component analysis to reduce the dimensions of the discovered facets. First, we examined the interitem correlations, the vast majority of which were above the .3 threshold. Second, we employed the Kaiser-Meyer-Olkin measure of sampling adequacy, resulting in satisfactory values (.83 and .70) above the 0.5 threshold for alpha and beta dimensions, respectively. Next, we estimated the internal consistency using Cronbach α, which resulted in satisfactory coefficients of .92 and .80, respectively. For further analysis, we retained four components for each of the two dimensions with eigenvalues greater than 1 (alpha: 8.4, 4.1, 1.7, and 1.2 account for 86% of variation; beta: 4.2, 2.6, 2.1, and 1.2 account for 83% of variation).

Table 3 presents the definitions and descriptive statistics of the variables.

**Table 3:** Descriptive statistics (N=2891).

Variables		Mean (SD)	Minimum	Maximum
**Dependent variable**				
	Fatal opioid overdose (lower)	10.6 (36.8)	0	972
	Fatal opioid overdose (upper)	17.6 (34.9)	9	972
**Independent variables**				
	Openness	74.8 (6.88)	0	100
	Conscientiousness	78.2 (6.48)	0	100
	Extraversion	19.4 (4.28)	0	100
	Agreeableness	33.8 (3.72)	0	100
	Neuroticism	44.0 (5.75)	0	100
**Control variables**				
	Age-adjusted years of potential life lost rate per 100,000	7994 (2306)	2397	23,850
	Births with low birth weight (<2500g; %)	8.2 (2.0)	2.8	18.8
	Adults who reported BMI≥30 (%)	30.8 (4.3)	12	48.1
	Indicator of access to healthy foods: 0 is worst, 10 is best	7.2 (1.0)	0	10
	Adults who report no leisure time physical activity (%)	27.4 (545)	9.2	44.9
	Population with access to places for physical activity (%)	59.6 (23.2)	0	100
	Driving deaths with alcohol involvement (%)	31.2 (13.7)	0	100
	Sexually transmitted disease (chlamydia cases/population per 100,000)	355.8 (246.7)	34.7	2854.3
	Teen births/females aged 15-19 years per 1000	43.4 (19.3)	3.7	130.4
	Population younger than 65 years without insurance (%)	17.4 (5.3)	2.9	39.5
	Discharges for ambulatory care sensitive conditions/Medicare enrollees per 1000	69.9 (27.7)	153.9	280.6
	Diabetic Medicare enrollees receiving HbA_1c_^a^ test (%)	84.3 (6.0)	17.5	97.3
	Female Medicare enrollees having at least one mammogram in 2 years (age 67-69; %)	60.8 (8.0)	24.1	84.6
	Adults aged 25-44 years with some postsecondary education (%)	55.3 (11.3)	18.7	88.3
	Population 16 years or older that are unemployed and looking for work (%)	7.2 (2.5)	0.8	28.2
	Children (younger than 18 years) living in poverty (%)	24.4 (9.1)	3.3	65.9
	Children living in single-parent households (%)	32.8 (9.7)	0.6	78.6
	Households with at least one of four housing problems: overcrowding, high housing costs, lack of kitchen, or lack of plumbing facilities (%)	14.5 (4.3)	4.2	52.4
	People who drive alone to work (%)	79.6 (6.0)	6.2	95.3
	Among workers who commute in their car alone, those that commute more than 30 minutes (%)	30.4 (11.8)	0.3	71.2
	Words contained in aggregated tweets by county (language control variable)	7364 (11,974)	104	46,560

^a^HbA_1c_: glycated hemoglobin.

### Econometric Model Specification

Our estimation method is based on the nature of our dependent variable. Given the privacy constraints resulting in data suppression when the number of reported deaths is less than 10, we decided to treat the missing observations in the outcome as an *interval* censored between zero and nine. Therefore, for an interval type of outcome, we chose a linear regression model with panel-level random effects to test our hypotheses. Note, the fixed-effects specification was not feasible for this because Stata’s -xtintreg- command (which we used for estimation) relies on Gauss-Hermite quadrature to estimate the likelihood function, which ultimately keeps the locations and weights of clusters fixed during optimization. To at least partially adjust for this effect, we accounted for yearly fixed effects by including year dummies in the model.

*y_it_* = *X_it_β* + *C_it_β* + *υ_i_* + *ε_it_*     **(1)**

for *i* = 1,...,*n* counties, where *t*= 1,...,*n_i_*; *y_it_* is the outcome of interest; *X_it_* is a vector of focal regressors corresponding to the Big Five personality traits; *C_it_* is a vector of observed controls; *υ_i_* is a random effect; and *ε_it_* is the error term. The observed data consist of the pairs (*y*_1_*_it_*, *y_2it_*), such that *y_1it_*≤*y_it_*≤*y_2it_*, where *y_1it_* is 0 and *y_2it_* is possibly +∞. To account for yearly fixed effects, we added year dummies in the estimated models.

Although we made a significant effort to control for observed confounders, there might still be endogeneity caused by omitted variable bias. For example, cognitive abilities [91] and cultural norms [92] are likely to be correlated with the Big Five and to affect drug overdose behavior. To alleviate omitted variable bias concerns, given the nonlinear outcome distribution and the continuous nature of the endogenous Big Five, we used a control function method [93]. First, we needed instruments that are theoretically associated with the personality traits but not with the error term (*ε_it_*) in fatal opioid overdoses. Relatedly, the prior literature has emphasized that personality traits are associated with aspects of natural language use and linguistic styles [82,94] as well as grammar and punctuation [95,96]. Neither of these factors is directly related to the behavior leading to drug overdose. We therefore identified multiple language-related characteristics as candidates for instrumental variables. To extract linguistic and writing characteristics from tweets, we used an advanced text analysis application called LIWC2015 [83]. We obtained a set of 46 characteristics (eg, analytic, clout, tone, six-letter words, words per sentence, nouns, verbs, and punctuation). For further analysis, we selected only those measures that were correlated moderately or strongly with the corresponding personality traits and weakly with the outcome, and significantly predicted the corresponding personality traits (Table A1 in Multimedia Appendix 1). Second, to estimate the first-stage residuals, we regressed each of the Big Five personality traits on the selected sets of instruments, *Z_it_*, including all second-stage observables (*C_it_*):

*X_it_* = *Z_it_β* + *C_it_β* + *α_i_* + *ε_it_*     **(2)**

To ensure the validity of our instruments, we further subjected them to a series of weak identification tests. The results are summarized in Table 4.

Our test results provide suggestive evidence in favor of the validity of the selected instruments and, therefore, the plausibility of our endogeneity correction strategy. Therefore, to correct for omitted variable bias in equation 1, we included the first-stage residuals (denoted *R_it_*) obtained from equation 2.

**Table 4:** Weak identification tests of the instrumental variables.

Variables	Anderson underidentification test, *P* value	Stock-Yogo weak-identification test (5%)	Sargan overidentification test, *P* value	Davidson-MacKinnon test of endogeneity, *P* value
Openness	<.001	21.0	.31	.35
Conscientiousness	<.001	20.9	.30	.59
Extraversion	<.001	19.8	.28	.14
Agreeableness	<.001	18.4	.26	.07
Neuroticism	<.001	20.7	.38	.19

## Results

The results of our analysis reveal several insights, including a counterintuitive finding. In Table 5, we present our estimates obtained across several models. First, to establish a baseline for model fit assessment, we proceeded by introducing our control variables only (model 1; refer to part B of Multimedia Appendix 1 for more details related to the selection of the control variables). Second, we included the main effects (model 2) followed by the main effects and control function (model 3) models. Additionally, in Multimedia Appendix 1, we include several models with alternative specifications to account for outcome variable distribution, imputation bias, and additional observed confounders (models 4-6, respectively). These models ensure consistency of our estimates and robustness of our modeling approach.

**Table 5:** Panel interval regression models of fatal opioid overdose (bootstrapped SEs).

Variables	Model 1		Model 2		Model 3^a^	
	Treatment effect, β (SE)	*P* value	Treatment effect, β (SE)	*P* value	Treatment effect, β (SE)	*P* value
Years of potential life lost rate	–.001 (.0001)	<.001	–.001 (.0001)	<.001	–.001 (.0001)	<.001
Low birth weight (%)	.946 (.185)	<.001	.934 (.176)	<.001	.970 (.221)	<.001
Adult obesity (%)	–.597 (.074)	<.001	–.596 (.085)	<.001	–.638 (.139)	<.001
Food environment index	3.235 (0.655)	<.001	3.184 (0.873)	<.001	4.606 (3.127)	.16
Physically inactive (%)	–.130 (.084)	.12	–.128 (.071)	.07	–.076 (.107)	.48
Access to exercise opportunities (%)	.077 (.011)	<.001	.076 (.001)	<.001	.072 (.019)	<.001
Alcohol-impaired driving deaths (%)	.009 (.013)	.47	.012 (.015)	.43	.014 (.017)	.42
Sexually transmitted infections rate	.008 (.001)	<.001	.008 (.001)	.43	.008 (.002)	<.001
Teen birth rate	.033 (.028)	.24	.041 (.029)	.16	.144 (.191)	.45
Uninsured (%)	–.311 (.128)	.02	–.332 (.154)	.03	–.232 (.293)	.43
Preventable hospital rate	.040 (.008)	<.001	.040 (.011)	<.001	.057 (.035)	.10
Diabetic monitoring (%)	.015 (.037)	.69	.017 (.041)	.68	.003 (.045)	.96
Mammography screening (%)	.041 (.027)	.14	.035 (.027)	.20	.039 (.034)	.24
Some college (%)	.335 (.037)	<.001	.336 (.038)	<.001	.338 (.057)	<.001
Unemployed (%)	–.265 (.097)	.01	–.285 (.124)	.02	–.016 (.594)	.98
Children in poverty (%)	.171 (.068)	.01	.167 (.075)	.03	.204 (.098)	.04
Single-parent households (%)	.142 (.035)	<.001	.144 (.037)	<.001	.133 (.063)	.04
Severe housing problems (%)	1.151 (0.130)	<.001	1.162 (0.179)	<.001	1.232 (0.133)	<.001
Driving alone to work (%)	–.416 (.120)	.001	–.419 (.135)	.002	–.500 (.166)	.003
Long commute–drives alone (%)	.338 (.048)	<.001	.336 (.043)	<.001	.292 (.077)	<.001
Word count (language control variable)	.001 (.0001)	.01	.001 (.0001)	.05	.001 (.0001)	.07
Alpha component 1	.046 (.386)	.91	–.060 (.418)	.89	–.213 (.484)	.66
Alpha component 2	–2.088 (.342)	<.001	–2.102 (.383)	<.001	–2.183 (.389)	<.001
Alpha component 3	1.565 (.472)	.001	1.569 (.471)	.001	1.822 (.595)	.002
Alpha component 4	.259 (.192)	.18	.304 (.231)	.19	.365 (.250)	.14
Beta component 1	.809 (.468)	.08	.893 (.509)	.08	.999 (.555)	.07
Beta component 2	–1.150 (.276)	<.001	–1.279 (.276)	<.001	–1.372 (.330)	<.001
Beta component 3	.700 (.408)	.09	.733 (.366)	.05	.866 (.429)	.04
Beta component 4	1.992 (0.371)	<.001	2.049 (0.382)	<.001	2.165 (0.340)	<.001
Openness	N/A^b^	N/A	.049 (.049)	.32	.060 (.060)	.31
Conscientiousness	N/A	N/A	.229 (.056)	<.001	.243 (.061)	<.001
Extraversion	N/A	N/A	.308 (.076)	<.001	.331 (.098)	.001
Agreeableness	N/A	N/A	–.048 (.060)	.42	–.060 (.064)	.35
Neuroticism	N/A	N/A	.248 (.063)	<.001	.261 (.057)	<.001
Year dummies	Yes	N/A	Yes	N/A	Yes	N/A
First-stage residuals (control function)	No	N/A	No	N/A	Yes	N/A
Observations, n	8317	N/A	8278	N/A	7809	N/A
Counties, n	2891	N/A	2884	N/A	2717	N/A
Akaike information criterion	45,123.1	N/A	44,992.4	N/A	43,157.9	N/A
Bayesian information criterion	45,362.0	N/A	45,266.3	N/A	43,464.3	N/A

^a^Since residuals are estimated for each of the Big Five, there are differential patterns of missing values that ultimately result in missing values when added in model 3.

^b^N/A: not applicable.

*Hypothesis 1* predicts a significantly positive impact of openness on fatal opioid overdose. However, the results of our main (conservative) analysis reveal an insignificant relationship (*P*=.32). Although the coefficient is not significant, the sign is positive, as we hypothesized. This is consistent with prior literature that shows a positive relationship between openness and substance dependence [40,41] and use [9,11,12,48]. Although different OCEAN traits uniquely influence substance use, and different substances account for different levels of intensity of each personality trait, extant literature consistently reports the presence of high impulsivity and sensation-seeking in the personality profiles of substance users [97]. This finding is particularly relevant to openness, since the central facet of this trait is being open to new experiences and an elevated willingness to try new things—markers of impulsivity. Therefore, despite the lack of statistical significance for this indicator, we caution clinicians and public health experts in counties with high prevalence of this psychological trait to be mindful of the correlation between certain dimensions of openness and substance use. Clinicians in regions with higher levels of openness may need to engage in more limit-setting counseling and institute more intensive screening practices to monitor opioid use.

In *hypothesis 2*, we hypothesized a negative effect of conscientiousness on the outcome. Surprisingly, however, the coefficient is significantly (and consistently) positive (*β_Conscientiousness_*=.229, *P*<.001), contrary to both *hypothesis 2* and prior literature demonstrating the negative effect of conscientiousness on multiple types of substance use disorders such as alcohol abuse and dependence [12,44,46], longitudinal substance use [9,40], and drug use [11,48]. *Hypothesis 2* is therefore not supported. Given the consistent findings regarding the negative relationship between conscientiousness and substance use in the medical and psychological literature, rather than undermining the robust theoretical link between the two constructs in search of a plausible explanation for the counterintuitive result for conscientiousness, it is more helpful to explore the operationalization of this variable in greater detail instead. A closer look at the lexicon-based personality inference model reveals, for instance, that whereas the average number of LIWC categories associated with each of the five traits in the FFM is 21, only 15 categories significantly correlate with conscientiousness [14]. This peculiarity suggests that this personality trait may not lend itself to measurement with a psycholinguistic dictionary as well as the other four traits. To further investigate this issue, we used an alternative, open vocabulary, big data approach for personality trait inference implemented by the IBM Watson “Personality Insights” service [90]. Interestingly, despite their computational differences—one using LIWC and the other a global vectors approach for word representation (the GloVe word embedding technique)—both operationalizations of OCEAN show a positive sign for the effect of conscientiousness on fatal opioid overdose. Given a mean conscientiousness value of .3 for the alternative operationalization, it is plausible to assume that the model is not very confident in determining whether someone should be attributed this personality trait or not. These findings point to the need for a critical examination of the way linguistic methods for personality trait inference operationalize the construct of conscientiousness. Although we would expect higher levels of conscientiousness to be associated with fewer fatal overdose cases due to the consistent negative relationship between conscientiousness and substance use in the extant literature, our results indicate high concentrations of this psychological trait may point to higher opioid overdose mortality. More work may be needed to help clinicians in counties with particularly high levels of conscientiousness implement specific communication strategies designed to engage in problem solving in the context of pain and opioid therapy. For instance, one of the facets of conscientiousness that may be related to opioid mortality is a persistence-like factor, perseverance, which is not confined to this personality trait alone but rather overlaps with neuroticism, a known risk factor for substance use [98]. It is therefore plausible to expect that areas where this dimension of conscientiousness is elevated might benefit from focused habit-breaking counseling such as cognitive behavior therapy techniques.

*Hypotheses 3a* and *3b* are a set of competing (positive and negative) hypotheses, which account for the conflicting empirical evidence provided in the existing literature about the relationship between extraversion and substance use. Our model shows a positive statistically significant coefficient for extraversion (*β_Extraversion_*=.308, *P*<.001), thus supporting *hypothesis 3a.* This result suggests that, approximately for a 3-unit increase (percent) in the relative standing of a county on neuroticism, the expected number of overdoses increases by 1 death. Similar positive correlations for extraversion have been found in the context of alcohol use disorder, dependence, and abuse [12,44], as well as first-time and subsequent illicit drug use [48]. A possible explanation for this relationship could be the underlying factors of this personality trait, including high energy and high preference for excitement and stimulation, personality dimensions that have consistently been found to be related to addiction [97]. To curb opioid mortality in regions with high levels of extraversion, it might be beneficial to engage in problem-solving and limit-setting therapeutic techniques of the kind suggested for high-openness individuals.

Based on *hypothesis 4*, agreeableness will have a significantly negative effect on the outcome. However, our main (conservative) results previously presented demonstrate insignificant impact (*P*=.42). Consistent with the literature [6,40,41,48,71] and our expectations, the direction of the effect is negative. Although the results of our main analysis presented here do not show a statistically significant effect, the results of our robustness analysis (Table B1, Multimeida Appendix B) provide suggestive evidence of the partial support that the effect of the agreeableness personality trait on fatal opioid overdose is significant (*P*=.04). The strong theoretical support for a negative relationship between agreeableness and substance use could be explained by the personality profile of individuals who score low on this trait: hostile, self-centered, and spiteful [6]. This profile correlates with recent findings about the positive association between opioid overdose deaths and high levels of anger [24]. Specifically, although anger and irritation are transient emotional states rather than stable personality traits, individuals experiencing these negative emotions frequently also exhibit a lower ability to regulate anger, a facet at the intersection of high neuroticism and low agreeableness [24]. Opioid mortality monitoring and prevention programs in areas with conspicuously low levels of agreeableness can benefit from developing psychological treatments focused on problem-solving techniques, self-expression, and anger management [24].

Finally, we observed a significantly positive impact of neuroticism on fatal opioid overdose (*β_Neuroticism_*=.248, *P*<.001). Approximately for a 4-unit increase (percent) in the relative standing of a county on neuroticism, the expected number of overdoses increases by 1 death. This result further corroborates a substantial body of empirical evidence in the psychology and medical literature demonstrating a positive relationship between neuroticism and substance use disorders [4,70,72], opioid abuse [73], nonmedical prescription drug use [47,74], and longitudinal pain and prescription opioid medication use [10]. A possible explanation for the robust positive association between high neuroticism and opioid mortality could be the composition of the dimensions comprising this construct, including impulsivity [6], a known risk factor for substance use that is stable across different types of substances [97]. Neurotic individuals tend to be more negativistic, avoidant, and emotionally labile, and exhibit negative affectivity [6,24]. High neuroticism is also related to low agreeableness through the facet of anger regulation, which was recently found to have a positive impact on opioid overdose deaths [24]. It can thus be beneficial for clinicians to closely monitor areas with this personality trait combination (high neuroticism and low agreeableness), as it may amplify the risk for opioid mortality. In terms of possible therapeutic interventions, it might be advantageous to emphasize the strengthening of coping skills and emotion regulation as part of opioid therapy.

## Discussion

### Theoretical and Practical Implications

Our study is part of a nascent stream of research in health informatics that combines geospatial information, medical data, and unstructured user-generated content used to infer community characteristics. In the context of the relationship between personality and opioid mortality specifically, our study demonstrates the relevance and usefulness of examining personality at the community level, also referred to as geo-personality [24]. This approach allows our personality trait predictor variables to more closely approximate the idiosyncratic nature of opioid deaths, which are known to cluster geographically, with the Midwest, Appalachia, and Northeast of the United States being particularly affected [24]. Personality traits have also been found to cluster geographically, which adds more face validity to our geospatial methodology [24]. The community-level geo-personality infoveillance technique used in this paper has several important implications for the theory and practice of health informatics.

Our model and findings address a gap in the literature, which has hitherto not considered the explanatory power of personality in opioid-related outcomes. Specifically, building on the theoretical foundations of the FFM, we provide a more nuanced understanding of how and to what extent openness, conscientiousness, extraversion, agreeableness, and neuroticism contribute to fatal overdose. This knowledge is important because the relative consistency of the five personality dimensions across individuals and groups (eg, communities or counties) provides a stable and detailed framework for the establishing of relationships and, consequently, prevention of such complex behaviors as opioid overdose. Interestingly, contrary to existing findings [70,71,74], agreeableness was shown to have a positive impact on fatal opioid overdose. This contradiction suggests a need for further examining the complex constructs of the Big Five personality factors in the context of health behaviors; although their underlying constituents show remarkable uniformity along other behavioral aspects, in health choices, these monolithic structures may exhibit internal divisions. Furthermore, although personality assessment has long been an important factor in the recruitment of medical personnel by medical schools and health care facilities, our study points to the importance of assessing personality tendencies among patients as well.

In light of the record-high budget allocations for opioid addiction countermeasures, our findings also contribute to practice and can be used for the purpose of developing actionable intervention plans on the part of local municipalities and health providers alike to prompt assessments of at-risk individuals in real time (and prior to prescription) as opposed to implementing impersonal *en masse* Naloxone programs; guidelines for design and implementation of psychometric segmentation strategies, a form of market segmentation that divides consumers into subgroups based on shared psychological characteristics, which can identify personality profiles and pivot to those most closely associated with drug use disorders; IT artifact creation to support health care providers’ decision making related to opioid prescribing; or cognitive psychological programs that complement treatment with medication. To ensure the practical value of our study, we contacted a medical practitioner, who proposed concrete ways in which the insights from our findings could directly benefit the health care field. In addition to the aforementioned applications, the expert suggested the use of our Big Five population assessment mechanism to potentially predict rates of neonatal abstinence (withdrawal) and to plan for county resources for rehabilitation of individuals with opioid use disorder. For instance, if a county exhibits high rates of “agreeableness” as reflected in Twitter data and the use of a validated screening tool, allocating resources for rehabilitation in that county prior to an overdose event could significantly impact the number of overdoses within it.

### Implications for Health Informatics

From a health informatics perspective, our research represents a novel approach in three ways. First, we demonstrate the feasibility of intelligently mining unstructured (Twitter) data for epidemiologic discoveries, eliminating the potential ethical dangers of privacy and confidentiality breaches by aggregating personality scores at the communal (county) level—a research technique with proven value in the epidemiology literature [92]. Second, we show that language used by Twitter users can provide cues associated with the Big Five personality traits at the county level. This addresses the limitations of assessment of such data using traditional approaches, which often have limited spatial and temporal precision [99]. Moreover, the use of a psycholinguistic approach for Big Five personality trait assessment allows for a level of model explainability, transparency, and replicability, which are not always possible with more complicated or proprietary “black-box” open-dictionary approaches based on deep learning techniques. Finally, given the fact that major opioid-related statistics are reported by counties and states with a time lag, analysis of readily available Twitter data allows us to overcome this limitation and provide up-to-date estimates of opioid-related outcomes.

### Conclusion and Limitations

We studied the impact of personality traits at the county level on fatal opioid overdose, a nationwide crisis. In particular, we used a FFM that included openness, conscientiousness, extraversion, agreeableness, and neuroticism dimensions. We used publicly available multisource data and operationalized our focal predictors using a robust lexicon-based implementation well established in the information systems literature. We tested our model using robust econometric modeling, accounting for endogeneity caused by omitted variable bias using an instrumental variable approach. Overall, our results obtained by means of Twitter mining are consistent with the prior literature, yet they suggest several surprising insights.

The study is not without limitations. First, given that we merged multiple data sets from different sources, some information was lacking for a number of counties due to missing values. Consequently, some descriptive statistics might differ from those in other published studies. Second, because of the “suppressed value” limitation on the outcomes, our results are an approximation, although they still provide reasonable estimations and useful inferences. Third, although one might prefer analysis that handles interval-censored count outcomes, we are not aware of any such analysis (including longitudinal data considerations). Fourth, admittedly, only a small fraction (less than 3%) of Twitter users report locations of their residence as well as the origin of the tweets, thereby limiting the representativeness of our sample. Fifth, we note that counties differ significantly in terms of area and population size, rurality, education, household income, power of county governments, and poverty rates, leading us to assume differences in the personality traits of the population in different counties. Yet, although we observed evidence showing that the personality scores of different counties vary, it only reflects the personality traits of the Twitter users who report their locations, thus leading to some selection bias. Finally, despite the fact that we aggregated individual tweets to capture personality traits of the population at the county level, individual tweets allow a maximum of 140 characters, which might erect barriers to understanding the personality traits of a person from such a short piece of text.

## Appendix

Multimedia Appendix 1 Supplementary material.

## Abbreviations

CDC: Centers for Disease Control and Prevention
CHRR: County Health Rankings and Roadmaps
FFM: five-factor model
FIPS: Federal Information Processing System
ICD-10: International Statistical Classification of Diseases and Related Health Problems, 10th revision
JSON: JavaScript Object Notation
LIWC: Linguistic Inquiry and Word Count
OCEAN: openness, conscientiousness, extraversion, agreeableness, and neuroticism
WONDER: Wide-Ranging Online Data for Epidemiologic Research
