# Supplementary material for: Exploring the Association Between the “Big Five” Personality Traits and Fatal Opioid Overdose: County-Level Empirical Analysis
Source: JMIR Ment Health. 2021 Mar 8;8(3):e24939. doi: 10.2196/24939 (PMC7985797; doi:10.2196/24939)
Supplement: Multimedia Appendix 1 [file mental_v8i3e24939_app1.docx]

# Appendix A

**Table A1.** In the following table we provide descriptive statistics of the 1) facets underlying alpha/beta factors (mined using IBM Personality Insights service), and 2) selected instrumental variables (mined using LIWC2015).

|  | Facet/language characteristic | Mean | SD | Min | Max |
| --- | --- | --- | --- | --- | --- |
| **Alpha** | Achievement striving | .4 | .2 | .01 | .99 |
|  | Cautiousness | .4 | .2 | .01 | .99 |
|  | Dutifulness | .3 | .2 | .01 | .99 |
|  | Orderliness | .4 | .2 | .01 | .99 |
|  | Self-discipline | .5 | .2 | .01 | .99 |
|  | Self-efficacy | .5 | .2 | .01 | .99 |
|  | Altruism | .4 | .2 | .01 | .99 |
|  | Cooperation | .3 | .2 | .01 | .99 |
|  | Modesty | .5 | .2 | .01 | .99 |
|  | Morality | .4 | .2 | .01 | .99 |
|  | Sympathy | .4 | .2 | .01 | .99 |
|  | Trust | .3 | .2 | .01 | .99 |
|  | Anger | .6 | .2 | .01 | .99 |
|  | Anxiety | .6 | .2 | .01 | .99 |
|  | Depression | .6 | .2 | .01 | .99 |
|  | Immoderation | .7 | .2 | .01 | .99 |
|  | Self-consciousness | .5 | .2 | .01 | .99 |
|  | Vulnerability | .6 | .2 | .01 | .99 |
| **Beta** | Adventurousness | .4 | .2 | .01 | .99 |
|  | Artistic interests | .5 | .2 | .01 | .99 |
|  | Emotionality | .5 | .2 | .01 | .99 |
|  | Imagination | .4 | .2 | .01 | .99 |
|  | Intellect | .5 | .2 | .01 | .99 |
|  | Liberalism | .5 | .2 | .01 | .99 |
|  | Activity level | .4 | .2 | .01 | .99 |
|  | Assertiveness | .5 | .2 | .01 | .99 |
|  | Cheerfulness | .7 | .2 | .01 | .99 |
|  | Excitement seeking | .6 | .2 | .01 | .99 |
|  | Friendliness | .6 | .2 | .01 | .99 |
|  | Gregariousness | .6 | .2 | .01 | .99 |
| **Linguistic dimensions,**  **grammar,**  **and punctuation**  **(instruments)** | Analytic | 52.9 | 18.7 | 1 | 99 |
|  | Clout | 54.1 | 13.4 | 1 | 99 |
|  | Authentic | 54.5 | 18.1 | 1 | 99 |
|  | Emotional one | 58.4 | 21.6 | 1 | 99 |
|  | Six-letter words | 15.7 | 4.2 | 0 | 80 |
|  | Function words | 43.2 | 6.9 | 0 | 75 |
|  | “I” | 6.1 | 2.1 | 0 | 33.3 |
|  | “We” | 0.7 | 0.6 | 0 | 15.9 |
|  | “You” | 2.6 | 1.3 | 0 | 50 |
|  | Auxiliary verb | 7.8 | 2.2 | 0 | 37.5 |
|  | Negation | 2.0 | 0.9 | 0 | 25 |
|  | Pronoun | 14.7 | 3.7 | 0 | 50 |
|  | Verb | 16.1 | 3.4 | 0 | 46.7 |
|  | Adverb | 4.9 | 1.6 | 0 | 25 |
|  | Dictionary words | 78.5 | 7.6 | 10 | 100 |
|  | Netspeak | 2.2 | 1.3 | 0 | 33.3 |
|  | Informal | 4.1 | 2.0 | 0 | 37.5 |
|  | Swearing | 1.2 | 0.8 | 0 | 25 |
|  | Comma | 2.2 | 1.4 | 0 | 25 |
|  | Apostrophes | 2.5 | 1.3 | 0 | 37.5 |
|  | Colon | 1.4 | 1.4 | 0 | 24.9 |
|  | All punctuation | 46.2 | 15.0 | 0 | 283.3 |
|  | Period | 6.5 | 2.9 | 0 | 113.3 |
|  | Question mark | 20.1 | 13.8 | 0 | 266.6 |
|  | Conjunctions | 3.9 | 1.2 | 0 | 28.6 |
|  | Other punctuation | 6.5 | 5.8 | 0 | 100 |

# Appendix B. Robustness Analyses

We conducted the following robustness analyses to ensure plausibility of our modeling approach. First, we estimated Equation 1 using *non-imputed* observed number of deaths as the outcome variable using Ordinary Least Squares model with fixed effects (OLS-FE). Although the sample size (and corresponding number of yearly observations per county) has decreased significantly (from 2891 to only 787 counties), the observed effects are largely consistent with those obtained in the main part of the analysis (Models 2-3).

Second, given that the number of overdoses is a *count* type of data, we used a Poisson regression model (for the original non-imputed outcome) with fixed-effects specification^[[1]](#footnote-1)^ and robust standard errors. Results remain consistent.

Finally, it shall be mentioned that we started off with an extensive list of the county characteristics (controls) our initial list of control variables. These include *percentage of adults who report fair or poor health, average number of reported physically unhealthy days per month, average number of reported mentally unhealthy days per month, percentage of adults who report currently smoking, percentage of adults who report excessive drinking, population to primary care physicians ratio, population to dentists ratio, population to mental health providers ratio, graduation rate, violent crimes rate, injury mortality rate, and air pollution*. However, there were a substantial number of randomly missing values for these variables that decreased our sample size by at least 600 observations (20%) and total number of observations by about 2500 (28%). Therefore, we excluded several variables the notably high percent (>20%) of missing values from our analysis. To account for consistency of our estimates controlling for other confounders, we estimate Model 6 “All controls” using all available data. Our results not only ensure consistency of the previously observed effects, but also provide partial support in favor of the previously unsupported Hypothesis 4.

**Table B1.** Alternative models of fatal opioid overdose

| Variables | OLS-FE  Treatment Effect β (SE) | *P* value | Poisson-FE  Treatment Effect β (SE) | | *P* value | All controls  Treatment Effect β (SE) | | *P* value | |  |
| --- | --- | --- | --- | --- | --- | --- | --- | --- | --- | --- |
| **Control variables used in Models 1-3** | |  | |  | | | |  | |  |
| Years of potential life lost rate | –.007 (.003) | .01 | –.001 (.001) | | .07 | –.001 (.0001) | | <.001 | |  |
| Low birth weight (%) | –8.085 (5.916) | .17 | –.168 (.082) | | .04 | .108 (.405) | | .79 | |  |
| Adult obesity (%) | –.670 (.858) | .44 | –.014 (.014) | | .33 | –.820 (.175) | | <.001 | |  |
| Food environment index | 19.304 (43.730) | .66 | .981 (.732) | | .18 | 4.410 (4.727) | | .35 | |  |
| Physically inactive (%) | –.548 (1.241) | .66 | .028 (.022) | | .21 | –.041 (.165) | | .80 | |  |
| Access to exercise opportunities (%) | –.363 (.202) | .07 | –.005 (.004) | | .21 | .101 (.029) | | .001 | |  |
| Alcohol-impaired driving deaths (%) | .644 (.239) | .01 | .009 (.003) | | .01 | .039 (.032) | | .21 | |  |
| Sexually transmitted infections rate | –.015 (.021) | .47 | –.001 (.001) | | .05 | .005 (.002) | | <.001 | |  |
| Teen birth rate | .178 (2.699) | .95 | .062 (.037) | | .09 | .156 (.243) | | .52 | |  |
| Uninsured (%) | .157 (3.211) | .96 | .051 (.050) | | .31 | –.446 (.408) | | .27 | |  |
| Preventable hospital rate | –.120 (.462) | .80 | .005 (.007 | | .48 | .103 (.048) | | .03 | |  |
| Diabetic monitoring (%) | –.067 (.795) | .93 | –.005 (.011) | | .64 | .033 (.074) | | .65 | |  |
| Mammography screening (%) | .289 (.474) | .54 | .001 (.007) | | .94 | –.067 (.065) | | .30 | |  |
| Some college (%) | .175 (.874) | .84 | –.030 (.015) | | .04 | .369 (.093) | | <.001 | |  |
| Unemployed (%) | 2.849 (7.534) | .71 | .130 (.104) | | .21 | –.338 (.821) | | .68 | |  |
| Children in poverty (%) | 1.018 (.664) | .13 | .015 (.008) | | .05 | .222 (.123) | | .07 | |  |
| Single-parent households (%) | –.293 (.740) | .69 | –.005 (.013) | | .68 | .025 (.094) | | .79 | |  |
| Severe housing problems (%) | –.500 (1.560) | .75 | .025 (.025) | | .36 | 1.406 (.198) | | <.001 | |  |
| Driving alone to work (%) | –3.684 (2.212) | .10 | .032 (.032) | | .04 | –.623 (.234) | | .01 | |  |
| Long commute—drives alone (%) | –.344 (1.220) | .78 | .022 (.022) | | .32 | .438 (.124) | | <.001 | |  |
| Word count (language control variable) | .0002 (.001) | .69 | .0001 (.0001) | | .52 | .001 (.0001) | | .01 | |  |
| Alpha component 1 | –5.552 (4.922) | .26 | –.037 (.070) | | .60 | –1.371 (.821) | | .10 | |  |
| Alpha component 2 | –4.721 (3.029) | .12 | –.042 (.041) | | .30 | –3.111 (.664) | | <.001 | |  |
| Alpha component 3 | 10.717 (4.469) | .02 | .054 (.046) | | .24 | 3.457 (1.043) | | .001 | |  |
| Alpha component 4 | .854 (2.233) | .70 | –.055 (.031) | | .08 | .708 (.357) | | .05 | |  |
| Beta component 1 | .850 (5.430) | .88 | –.051 (.067) | | .44 | 1.842 (.825) | | .03 | |  |
| Beta component 2 | –8.357 (3.259) | .01 | –.060 (.041) | | .14 | –2.208 (.593) | | <.001 | |  |
| Beta component 3 | 4.546 (4.020) | .26 | –.004 (.033) | | .92 | 1.600 (.772) | | .04 | |  |
| Beta component 4 | 7.690 (2.743) | .01 | .064 (.029) | | .03 | 3.776 (.793) | | <.001 | |  |
| year=2015 | 7.865 (5.030) | .12 | –.024 (.076) | | .75 | 3.813 (.843) | | <.001 | |  |
| year=2016 | 27.684 (11.132) | .01 | .254 (.148) | | .09 | 12.731 (2.890) | | <.001 | |  |
| Linear prediction (residual 1) | –4.206 (3.331) | .21 | –.616 (1.148) | | .59 | –.812 (.355) | | .02 | |  |
| Linear prediction (residual 2) | 1.482 (4.556) | .75 | 3.925 (1.928) | | .04 | –.192 (.509) | | .71 | |  |
| Linear prediction (residual 3) | –11.426 (4.771) | .02 | –.417 (1.022) | | .68 | –1.033 (.512) | | .04 | |  |
| Linear prediction (residual 4) | –5.077 (4.698) | .28 | –1.710 (.927) | | .07 | .319 (.603) | | .60 | |  |
| Linear prediction (residual 5) | 3.685 (4.247) | .39 | 2.300 (1.428) | | .11 | –.945 (.452) | | .04 | |  |
| **Independent variables used in Models 1-3** | | | | | | | | | |  |
| Openness | –1.432 (1.111) | .20 | –.185 (.279) | | .51 | .089 (.145) | | .54 | |  |
| Conscientiousness | 5.052 (1.046) | <.001 | .909 (.248) | | <.001 | .618 (.131) | | <.001 | |  |
| Extraversion | 2.749 (.973) | .01 | .432 (.189) | | .02 | .743 (.191) | | <.001 | |  |
| Agreeableness | –2.010 (1.113) | .07 | –.227 (.114) | | .04 | –.300 (.162) | | .07 | |  |
| Neuroticism | 3.310 (1.165) | .01 | .296 (.278) | | .29 | .629 (.142) | | <.001 | |  |
| **Control variables excluded from Models 1-3 due to substantial number of missing values** | | | | | | |  | |  |  |
| Percentage of adults who report fair or poor health (age-adjusted) |  |  |  | |  | .485 (.121) | | <.001 | |  |
| Average number of reported physically unhealthy days per month |  |  |  | |  | –.974 (.479) | | .04 | |  |
| Average number of reported mentally unhealthy days per month |  |  |  | |  | .174 (.472) | | .71 | |  |
| Percentage of adults who report currently smoking |  |  |  | |  | .298 (.093) | | .001 | |  |
| Percentage of adults who report excessive drinking |  |  |  | |  | –.121 (.096) | | .21 | |  |
| Population to primary care physicians ratio |  |  |  | |  | –.001 (.0001) | | .001 | |  |
| Population to dentists ratio |  |  |  | |  | –.001 (.0001) | | <.001 | |  |
| Population to mental health providers ratio |  |  |  | |  | .001 (.0001) | | .002 | |  |
| Graduation rate (cohort or averaged freshman) |  |  |  | |  | –.160 (.040) | | <.001 | |  |
| Violent crimes/population * 100,000 |  |  |  | |  | .039 (.005) | | <.001 | |  |
| Injury mortality rate per 100,000 |  |  |  | |  | .035 (.027) | | .19 | |  |
| Air pollution (average daily PM25) |  |  |  | |  | 1.003 (.627) | | .11 | |  |
| *Constant* | *305.718 (821.901)* | *.71* |  | |  | *8.733 (79.448)* | | *.91* | |  |
| *AIC* | *15,936.1* |  | *7,444.5* | |  | *34,386.5* | |  | |  |
| *BIC* | *16162.6* |  | *7,666.9* | |  | *34,710.3* | |  | |  |
| *Observations* | *1,851* |  | *1,675* | |  | *5,477* | |  | |  |

1. The interpretation of the Poisson regression coefficients is that for a 1-unit change in the predictor variable, the difference in the logs of expected counts changes by the respective regression coefficient. [↑](#footnote-ref-1)
